# Comment on “Diversity Outbred: A New Generation of Mouse Model”

**DOI:** 10.1289/ehp.1510098

**Published:** 2015-09-01

**Authors:** Thomas C. Erren, Tracy E. Slanger, J. Valérie Groß, Russel J. Reiter

**Affiliations:** 1 Institut und Poliklinik für Arbeitsmedizin, Umweltmedizin und Präventionsforschung, Universitätsklinikum Köln, Köln, Germany; 2 Department of Cellular and Structural Biology, University of Texas Health Science Center at San Antonio, San Antonio, Texas, USA

A news article by Charles Schmidt propagates the Diversity Outbred mouse model to facilitate the extrapolation of toxicology findings to humans. In this regard, we would like to highlight two sources of animal diversity that could be relevant: *1*) the differential—and genetically codetermined—circadian propensity for activity and rest with which the mice are born, and *2*) the differential stability of circadian rhythms in later life stages of mice, to which perinatal photoperiods may contribute.

Regarding *1*), humans come in different chronotypes corresponding to variations in how physiology, endocrinology, metabolism, and behavior are organized and timed over the individual’s biological day and night. Inasmuch as the extent of genetic variability among model mice should be similar to genetic variations in humans, how do new-generation mouse models capture differential chronobiological propensity? Laboratory mice, like humans, come in various chronotypes, and different strains come in different ones (Wicht et al. 2014); therefore, different time windows of biological nights and days should significantly impact when and what we observe or measure, be it parameters of toxicology, behavior, physiology, or anatomy. With evidence that responses to DNA damage are regulated by the circadian clock in mice (Kang et al. 2010), chronotype-dependent lows and highs of DNA repair within a 24-hour period must be considered when extrapolating mouse-based toxicological data to humans.

Regarding *2*), although inbred mice may be genetically identical, perinatal photoperiods may nevertheless—by imprinting the circadian clock—lead to a differential stability of circadian rhythms. Perinatal exposures to summer versus winter light conditions (i.e., with a light:dark ratio of 16:8 versus 8:16) can determine the susceptibility of a mouse’s circadian rhythm to dysfunction or disruption throughout the animal’s life (Ciarleglio et al. 2011). Furthermore, the integrity of circadian clocks and rest–activity circadian rhythms plays a major role for tumor suppression by controlling cell proliferation and other cellular functions (Fu and Lee 2003). Taken together, if the perinatal photoperiod may codetermine the very robustness of mice to fight off severe circadian dysfunction (Filipski et al. 2002) and the development of tumors, then *a fortiori* we would have to understand, and possibly control, circadian diversity.

Overall, the biological activity of circadian clocks must be taken into account when experimenting with mice. Taking note of *1*) and *2*), we should consider possible chronotypes in new-generation mice and standards for the light:dark conditions under which laboratory mice are bred and raised. Moreover, the efficacy, toxicity, and carcinogenicity of chemicals or drugs should be tested at different times within a 24-hour period. Given evidence for links between aging clocks and progressive declines of the circadian control of crucial biological processes (Belancio et al. 2014), age should also be factored in when using mice for testing.
